# Editorial: Does selection against autoreactive B cells limit affinity maturation to pathogens?

**DOI:** 10.3389/fimmu.2023.1294532

**Published:** 2023-10-04

**Authors:** Marilyn Diaz, Laurent Verkoczy

**Affiliations:** Immunology and Vaccines Discovery Group, Applied Biomedical Science Institute, San Diego, CA, United States

**Keywords:** somatic hypermutation, B cells, autoreactive, affinity maturation, HIV-1, influenza, antibodies

The adaptive immune system evolved to respond to an unpredictable universe of pathogens by generating a large repertoire of lymphocytes. It accomplishes this through the mix and match of gene segments encoding the immunoglobulin and T cell repertoires during V(D)J recombination (1). It is estimated that V(D)J rearrangement can generate roughly 3×10^11^ specificities. However, novel specificities can also recognize the universe of self-antigens in organisms, a potential problem that can lead to autoreactivity and autoimmune diseases. The immune system largely keeps this problem in check by the elimination of strong autoreactivities during central tolerance in B cells, and in thymic development of T cells (2, 3). In B-cells, this pruning of autoreactive specificities is accomplished through a variety of mechanisms including deletion of autoreactive cells, inactivation of the cell responsiveness to activation (anergy), and swapping of the light chain (Receptor Editing) (2). Finally, by requiring multiple “danger” signals to initiate an immune response, the immune system is able to “tolerate” low affinity self-reactive cells whose deletion may lead to significant holes in the lymphocytic repertoire. These danger signals include the role of toll receptors that interact with pathogen debris during an infection to amplify activation signals (4). Thus, through a combination of somatic diversity-generating mechanisms, and checkpoints to prune autoreactive specificities, the vertebrate adaptive immune system has managed to strike a balance that yields an efficacious yet tolerant adaptive response against a sea of potential pathogens.

B cells undergo further diversification following activation by T-dependent antigens through somatic hypermutation (SHM). This process alters B cell receptors specificities by introduction of point mutations throughout the variable regions of the heavy and light chains (5, 6). SHM is coordinated with positive selection for increased affinity of B cell receptors in transient structures known as germinal centers (GC) that develop in secondary lymphoid organs during an immune response (7, 8). SHM is another opportunity for the generation of not just higher affinity receptors to foreign antigen but also of autoreactive B cell receptors that need to be purged from the memory B cell compartment. Because of the multitude of tolerance checkpoints to eliminate autoreactivities generated either during V(D)J recombination or during SHM in B cells, the existence of repertoire gaps or “holes” that inhibit immune responses to certain pathogens is very likely. Similarly, many pathogens exploit these repertoire holes through molecular mimicry, thus avoiding detection by adaptive immune cells (9). The notion that B cell receptors can mutate away from autoreactivity towards high affinity to foreign pathogens was initially proposed by Jerne (10), and later by Diaz and Klinman (11) but experimental evidence is needed. The focus of this Research Topic is to explore the extent by which autoreactivity and tolerance mechanism may prevent effective B cells responses in GC’s to important pathogens.

An insightful exploration of this Research Topic is provided by Young et al. In their review, multiple studies are summarized, including their own, that demonstrate this conflict is in play in GC’s and the outcome depends on whether self-reactivity and increased affinity can be decoupled allowing high affinity receptors to be “redeemed” and participate in the immune response. Furthermore, they describe evidence that when this decoupling is not achievable, the outcome of these ‘unredeemable’ B-cell specificities is their elimination from the repertoire (12). This is a key finding as it demonstrates that such a conflict can effectively inhibit the immune response to some pathogens.

By using site directed back mutation of the broadly neutralizing lineage, CH103-106, Li et al. provide experimental evidence that SHM controlling autoreactivity and neutralization can be decoupled, thus opening the door for the possibility that at least some bnAb immune responses directed to the CD4 binding site of HIV-1 are ‘redeemable, and could be manipulated by empirical or rational immunogen design to exploit this non-overlap between self and non-self. This is also consistent with the proposal (13) that autoreactivity decoupling from neutralization may potentiate more potent broadly neutralizing (bnAb) antibody responses to HIV-1, the prototype pathogen for demonstrating that autoreactive bnAb-expressing B-cells are culled by multiple tolerance controls (14, 15). Similar restrictions in antibody responses to certain antigens have also been shown to impede bnAb responses to influenza antigens (16).

The contribution by Denis-Lagache et al., explores the hypothesis that a mechanism known as locus suicide recombination (LSR) may contribute to the elimination of Activation-Induced deaminase (AID) secreting autoreactive B cells in GC’s is explored. LSR was previously shown by this group to occur from AID-mediated recombination following class switch recombination involving the 3’ cis regulatory region and resulting in the deletion of the constant domain cluster and loss of immunoglobulin receptor expression (17). This study examines whether LSR is an additional mechanism (besides apoptosis) of deletion of *de novo* autoreactive B cells during SHM in GC’s. Using a novel reporter mouse model and LSR-deficient mice, they provide evidence that LSR plays a role in GC negative selection and may further contribute to loss of important specificities during an immune response.

Finally, the paper by Petrova et al., examines the potential of GC B cells undergoing SHM but failed negative selection to become neoplastic and the relationship of the B cell repertoire to this outcome. SHM, V(D)J and CSR all involve deliberate DNA breakage which can lead to the formation of cancer cells. In this study, they make the interesting observation that the repertoire of transformed B cells in certain blood cancers is unique and different from similarly derived normal GC B cells.

Combined, these studies highlight the fine balance in GC B cells to obtain high affinity B cell receptors while eluding disease outcomes such as autoreactivity and cancer. This ongoing “tug of war” in germinal centers can also impede, the generation of an efficacious immune response to important pathogens. Overall, these studies not only highlight the problem but may offer ways to overcome barriers to efficacious B cells responses via approaches designed to decouple autoreactivity from neutralization of pathogens.

## Author contributions

MD: Conceptualization, Formal Analysis, Investigation, Project administration, Writing – original draft, Writing – review & editing. LV: Conceptualization, Formal Analysis, Funding acquisition, Resources, Supervision, Writing – original draft, Writing – review & editing.
